# Host-Dependent Differences in Replication Strategy of the *Sulfolobus* Spindle-Shaped Virus Strain SSV9 (a.k.a., SSVK1): Infection Profiles in Hosts of the Family Sulfolobaceae

**DOI:** 10.3389/fmicb.2020.01218

**Published:** 2020-07-14

**Authors:** Ruben Michael Ceballos, Coyne Gareth Drummond, Carson Len Stacy, Elizabeth Padilla-Crespo, Kenneth Mark Stedman

**Affiliations:** ^1^Department of Biological Sciences, The University of Arkansas, Fayetteville, AR, United States; ^2^Arkansas Center for Space and Planetary Sciences, Fayetteville, AR, United States; ^3^Cell and Molecular Biology Program, The University of Arkansas, Fayetteville, AR, United States; ^4^Departmento de Ciencias y Tecnología, Universidad Interamericana de Puerto Rico, Aguadilla, PR, United States; ^5^Department of Biology, Center for Life in Extreme Environments, Portland State University, Portland, OR, United States

**Keywords:** *Sulfolobus* spindle-shaped virus, lytic replication, allopatric evolution, sympatric coevolution, non-lytic release, *Sulfolobus*, fusellovirus, SSV9

## Abstract

The *Sulfolobus* Spindle-shaped Virus (SSV) system has become a model for studying thermophilic virus biology, including archaeal host-virus interactions and biogeography. Several factors make the SSV system amenable to studying archaeal genetic mechanisms (e.g., CRISPRs) as well as virus-host interactions in high temperature acidic environments. Previously, we reported that SSVs exhibited differential infectivity on allopatric vs. sympatric hosts. We also noticed a wide host range for virus strain SSV9 (a.k.a., SSVK1). For decades, SSVs have been described as “non-lytic” double-stranded DNA viruses that infect species of the genus *Sulfolobus* and release virions via budding rather than host lysis. In this study, we show that SSVs infect hosts representing more than one genus of the family *Sulfolobaceae* in spot-on-lawn “halo” assays and in liquid culture infection assays. Growth curve analyses support the hypothesis that SSV9 virion release causes cell lysis. While SSV9 appears to lyse allopatric hosts, on a single sympatric host, SSV9 exhibits canonical non-lytic viral release historically reported SSVs. Therefore, the nature of SSV9 lytic-like behavior may be driven by allopatric evolution. The SSV9-infected host growth profile does not appear to be driven by multiplicity of infection (MOI). Greater stability of SSV9 vs. other SSVs (i.e., SSV1) in high temperature, low pH environments may contribute to higher transmission rates. However, neither higher transmission rate nor relative virulence in SSV9 infection seems to alter replication profile in susceptible hosts. Although it is known that CRISPR-Cas systems offer protection against viral infection in prokaryotes, CRISPRS are not reported to be a determinant of virus replication strategy. The mechanisms underlying SSV9 lytic-like behavior remain unknown and are the subject of ongoing investigations. These results suggest that genetic elements, potentially resulting from allopatric evolution, mediate distinct virus-host growth profiles of specific SSV-host strain pairings.

## Introduction

In virology, *reduced model* systems are prokaryotic or single-cell eukaryotic viral systems that provide fundamental information regarding virus-host interactions and coevolution independent of the complexity found in macroorganisms with evolved immune systems. Reduced model viral systems have been used extensively to study fundamental properties of virus evolution (Lenski and Levin, 1985; Morgan et al., 2005; Brockhurst et al., 2007). Moreover, it is suggested that studying virus-host interactions in reduced model systems may provide opportunities to understand fundamental processes of virus evolution in host systems of agricultural or medical importance (Brockhurst et al., 2007; Dennehy, 2009). Given the recent view that the evolutionary origin(s) of viruses may be linked to the early evolution of Archaea (Forterre, 2006; Berliner et al., 2018) and the suggestion that the emergence of viruses likely pre-dates the divergence of the Archaea and Eukarya (Prangishvili et al., 2017; Krupovic et al., 2018), a robust archaeal virus model system could provide new insights into the evolution of virus lineages and viral replication strategies as well as mechanisms of viral virulence, host resistance, and virus attenuation.

The *Sulfolobus* Spindle-shaped Virus (SSV) system has become a popular model for studying thermophilic archaeal virus biology and virus-host biogeography. Several factors make this system ideal for studying virus-host infections in crenarchaea (i.e., Sulfolobales). First, endemic populations of SSV hosts from the family *Sulfolobaceae* exhibit biogeographic structure such that there is a positive correlation between genetic distance among strains (i.e., divergence) and geographic distance among various sites from which strains have been isolated (Grogan, 1989; Whitaker et al., 2003; Reno et al., 2009). SSVs also exhibit biogeographic structure on a global-scale (Held and Whitaker, 2009). Second, the highly acidic (pH < 4.5) and high temperature (65–88°C) SSV-*Sulfolobus* habitats have low biodiversity, limiting the potential for host switching, which can confound efforts to elucidate the genetic underpinnings of virus-host infection profiles (Munson-McGee et al., 2018). Third, SSVs and Sulfolobales can be readily cultured both in liquid media (e.g., yeast-sucrose, tryptone) and on gellan gum (e.g., Gel-Rite®) plates (Zillig et al., 1996; Stedman, 2008; Ceballos et al., 2012). Fourth, given the wide geographical separation between sulfuric hot springs, which are habitats for SSVs and hosts, this system is amenable to studying multiple allopatric and sympatric virus-host pairs (Ceballos et al., 2012), which is essential for studying virus-host interactions, biogeography, and coevolution (Greischar and Koskella, 2007).

SSVs comprise the *Fuselloviridae* (International Committee on Taxonomy of Viruses, 2017) with SSV1 being the founding archetype of this family of “little spindle” (*Latin: fusello*) shaped viruses (Martin et al., 1984; Reiter et al., 1987; Palm et al., 1991). SSV1 was isolated from *Sulfolobus shibatae* strain B12, which was derived from a sulfuric hot spring in Beppu, Japan (Martin et al., 1984; Grogan et al., 1990). The SSV1 virion was shown to harbor a positively-supercoiled double-stranded (ds) DNA genome (Nadal et al., 1986) and virion production was shown to be UV inducible (Martin et al., 1984; Schleper et al., 1992). Studies also demonstrated that, apart from the fully assembled virion, SSV1 could reside either episomally, as a positively-supercoiled, negatively-supercoiled, or relaxed dsDNA viral genome within the host (Nadal et al., 1986); or, fully integrated (often in tRNA genes) in the host genome as a provirus (Reiter et al., 1989).

Soon after the initial characterization of SSV1, other SSVs were isolated and characterized from geothermal regions worldwide. Until two recent publications that expand the number of characterized genotypes (Pauly et al., 2019; Zhang et al., 2020), only ten SSVs had been well-characterized with six of these strains: SSV1, SSV2, SSV3, SSV8 (a.k.a., SSVRH), SSV9 (a.k.a., SSVK1), and SSV10 (a.k.a., SSVL1) being the most extensively studied (for review see Goodman and Stedman, 2018). These SSVs have genomes that range from about 14.7–17.5 kbp (depending on the strain) consisting of 32–36 open-reading frames (ORFs) featuring polycistronic transcription units. Assembled SSV particles have a major axis of ~80–100 nm and a minor axis of ~60 nm.

For over three decades, SSVs have been reported to be non-lytic *budding* viruses that infect *Sulfolobus* (Martin et al., 1984; Reiter et al., 1987; Palm et al., 1991; Schleper et al., 1992; Zillig et al., 1998; Wiedenheft et al., 2004; Contursi et al., 2006; Prangishvili et al., 2006; Ceballos et al., 2012; Fusco et al., 2015; Quemin et al., 2016). The term *non-lytic* refers to the fact that SSV infection results in inhibition of cell growth (both in liquid culture and on lawns) rather than the gross lysis of host cells and cell death in liquid culture or clear plaques on host lawns—both of which result from lytic replication. Recently, it was suggested that SSV9 can induce a state of dormancy, empty cells, and eventual host death in a sympatric host; however, the mechanism for this proposed dormancy is unclear and there does not seem to be any cell lysis (Bautista et al., 2015).

Plaque-like “halo” assays using both sympatric and allopatric hosts have repeatedly shown that different SSVs (e.g., SSV1, SSV2, SSV3, SSV8, and SSV10) form turbid areas of growth inhibition or *halos* on host lawns (**Figures 2A, B**), often featuring diffuse boundaries (Martin et al., 1984; Schleper et al., 1992; Wiedenheft et al., 2004; Ceballos et al., 2012; Iverson and Stedman, 2012). Yet, halo assays using SSV9 (formerly known as SSVK1), isolated from the Valley of Geysers (*Russian*: Долина гейзеров) Kamchatka, Russia (Wiedenheft et al., 2004), form large *clear* plaques on host lawns (**Figure 2C**) in contrast to the *turbid* diffusely-bound halos characteristic of all other SSVs (Ceballos et al., 2012), indicating that SSV9 may replicate differently than the other SSVs (Bautista et al., 2015).

To test the hypothesis that SSV9 lyses susceptible hosts within the family *Sulfolobaceae*, host growth profiles in single-virus/single-host infection assays using three distinct SSVs (i.e., SSV1, SSV8, and SSV9) were evaluated. Liquid culture and *spot-on-lawn* “halo” assays were used to verify productive infection by a given SSV. To assess the relative susceptibility of different hosts to a given SSV and to elucidate conspicuous differences in host growth profiles during the infection, both allopatric and sympatric hosts were infected. Each specific virus-host pairing was conducted under different culture conditions to evaluate the impacts of distinct conditions on growth dynamics. For example, different multiplicities of infection (MOI) were tested and end-point assays (in liquid culture) were conducted to measure virus and host dynamics in parallel. In addition to evaluating halo “phenotypes” in plate-based assays, small-scale liquid culture assays were conducted to gauge the relative amount of cellular debris emerging from different virus-host pairings. Assays to assess the relative stability of different SSVs under conditions that simulate the natural Sulfolobales habitats were also performed to determine if virion stability plays a role in successful transmission and between strain differences in virion production.

## Materials and Methods

### Virus Preparation

Glycerol stocks of SSV-infected *Sulfolobus* strains stored at −80°C were partially thawed on ice. For most trials, 100 μL of infected cell suspension were added to 30 mL of YS media (pH 3.2) in a 125 mL Erlenmeyer flask (per Ceballos et al., 2012). Tryptone (T) or tryptone-sucrose (TS) media was used in select experiments by substituting 3.0 g L^−1^ tryptone for yeast and sucrose or 2.0 g L^−1^ tryptone plus 1.0 g L^−1^ sucrose for yeast, respectively. Flasks were loosely capped and incubated at 78°C/90 RPM shaking.

Once liquid culture reached an optical density (OD_600_) between 0.4 and 0.6, 3.0 mL of cell suspension was used to inoculate 600 mL of pre-heated fresh media in a 1.0 L baffled flask.

These cultures were incubated at 78°C/70 RPM until reaching an OD_600_ = 0.6–0.8 to maximize virus yields. The culture was subsequently centrifuged for 20 min at 6,000 RPM (Sorvall RT Legend Centrifuge, Fiberlite 4 × 800 mL fixed-angle rotor with 250 mL inserts; ThermoFisher, Pittsburgh, PA) to pellet the cell mass while leaving virus in suspension. The supernatant was decanted and filtered through a 0.45 μm vacuum PES filter system. The filtrate was concentrated using 10 kDa Centricon Plus-70 spin concentration tubes (Millipore Corp., Billerica, MA, USA) to produce ~3.0 mL concentrated SSV suspension.

TEM was used to confirm the presence of virions and virus particle count was measured using electrospray ionization/mass spectrometry or serial dilution halo assays (see below). Virus was stored at 4°C and used in *spot-on-lawn* “halo” assays and liquid culture infection assays within 2–3 weeks of harvesting. Dilutions of viral stocks were used as inocula.

### Host Cell Preparation

Glycerol stocks of uninfected *Sulfolobus* strains stored at −80°C were used to establish 30 mL cultures in YS (or T or TS) media, as described above. Uninfected *Sulfolobus* cultures were either used to prepare host lawns for halo assays (Ceballos et al., 2012) or used in liquid culture infection assays (as described below).

### Transmission Electron Microscopy (TEM) of Viruses and Cells

To verify the presence of SSVs in samples, ~5 μL of viral suspension was spotted onto a formvar-coated copper grid and incubated for 10 min in a humidity chamber. The sample was rinsed with distilled water and negatively stained with a 1% solution of uranyl acetate for 30 s. The stain was wicked off the grid and then the sample was air dried. Grids were imaged with a Hitachi H-7100 TEM at 75 kV. Images were captured at 60,000–200,000× magnification. TEM images for cells were acquired as described in Brumfield et al. (2009). Cells were fixed in glutaraldehyde (3% v/v), centrifuged, and re-suspended in a small volume of agar (2% v/v). After solidification, the resulting agar was cut into small pieces and fixed overnight with glutaraldehyde (3% v/v) in 0.05 M potassium sodium phosphate buffer (PSPB) and pH = 7.2. Agar pieces were rinsed twice for 10 min each with PSPB. Agar pieces were post-fixed with osmium tetroxide (2% v/v) at RT for 4 h. Samples were dehydrated via an ethanol rinse series (50%-100% v/v) and then washed with a transitional solvent, propylene oxide. Spurr's resin (Spurr, 1969) was used to infiltrate dehydrated cell mass and pieces were baked overnight at 70°C. Thin sections (60–90 nm) were cut and stained with uranyl acetate and lead citrate. Imaging was done with a LEO 912AB TEM.

### Electrospray Ionization/Mass Spectrometry (ESI/MS)

Virus suspension preparations were processed in an Integrated Viral Detection System (BVS, Inc.; Missoula, MT) per protocols in Wick and McCubbin (1999). A mixture consisting of 100 μL viral suspension and 900 μL ammonium acetate buffer solution is aerosolized in a Electrospray Aerosol Generator (Model 3480, TSI, Inc., Minnesota). A Differential Mobility Analyzer (Model 3081, TSI, Inc., Minnesota) separates particles by their electrical mobility, which is influenced by particulate mass-to-charge ratio or “size.” These particles flow in tandem with a saturated butanol fluid. The particles initiate butanol condensation and the stream is cooled enabling butanol-condensed particles to be optically counted in a Condensate Particle Counter (Model 3772, TSI, Inc., Minnesota). System software displays results in terms of particle count per size category with a standard range of 2–280 nm.

This specialized ESI/MS is designed to detect intact virus particles (Wick et al., 2006) and can measure relative virus particle count between two or more samples. In these infection assays, ESI/MS spectra were evaluated by using either peak values or the area under the curve to compare SSV particle production between two cultures.

Note: Time-of-flight estimation based on mass: charge is used to calculate ESI/MS “size.” ESI/MS “size” will differ from physical size as measured by other methods, such as transmission electron microscopy. In addition, mass:charge size vs. physical size will differ due to the assumption of virus particle sphericality in time-of-flight derivations. SSVs have been shown under electron microscopy to be fusiform or “spindle-shaped” particles of ~60 × 90 nm (and not spherical), thus, a discrepancy is expected. In some cases, SSVs exhibited dual peaks or shouldered peaks in the ESI/MS analyses. Such spectral features may be due to different conformations of the same SSV or, potentially, due to the presence of pleomorphic SSVs. Under ESI/MS, SSVs exhibit characteristic peaks between 46.1 and 61.5 nm for purified virus suspensions with major peaks typically appearing at 46.1, 47.8, and 49.6 within the ±4 nm system tolerance.

### Spot-on-Lawn Halo Assays

Halo assays were performed (per Stedman et al., 2003) and cultures were grown as described above. At an OD_600_ = 0.4–0.6, 500 μL of cell suspension were added to 4.5 mL of a 78°C mixture of equal parts 1.0% w/v Gelrite® (Sigma-Aldrich, St. Louis, MO, USA) and 2-fold concentrated YS medium. The 5 mL mixture was spread on pre-warmed Gelrite® plates (1% w/v in medium) and allowed to solidify for 15 min at room temperature followed by a 20 min incubation at 78°C. 1.0 μL of each viral suspension was spotted onto the prepared plate in labeled areas. Depending upon host strain, plates were incubated for 3–9 days at 78°C. Successful infection was scored by the formation of a visible *halo* of growth inhibition on the host lawn. Triton X-100 (0.05% v/v) was used as a positive control and sterile water was used as a negative control. Initial tests also used a 2 μL spotting of ultrafiltrate (10 kDa MWCO) to ensure that effects were not from other proteinaceous toxins (e.g., sulfolobicins) but from SSV infection.

### Liquid Culture Infection Assays

3.0 mL of uninfected *Sulfolobus* culture were diluted 1:100 in pre-heated media in a 1.0 L baffled flask. Cultures were grown to an OD_600_≈ 0.15. Then, 100 μL of standardized viral suspension were added to the flask. *Sulfolobus* cultures were incubated with shaking (78°C/70 RPM) for various time intervals. Virus was then harvested as detailed above and ESI was used for virus particle counts. Growth curves from SSV-*Sulfolobus* cultures were fit to Logistic, modified Logistic (Equations 1, 2), Gompertz, modified Gompertz (Equations 3, 4), and other growth models (Gompertz, 1825; Laird, 1965; Zwietering et al., 1990; Lopez et al., 2004; Sprouffske and Wagner, 2016):

(1) $$\begin{array}{r} y\left(t\right)=\frac{A}{1+{\left(\frac{{A-y}_{0}}{y_{0}}\right)e}^{-rt}} \end{array}$$

(2) $$\begin{array}{r} y\left(t\right)=\;\frac{A}{1+\exp\left(\frac{4\mu }{A}\left(\lambda -t\right)+2\right)} \end{array}$$

(3) $$\begin{array}{r} y\left(t\right)=\;A\cdot \exp\left[-\ln\left(\frac{y_{0}}{A}\right)\cdot \exp\left(-\mu \cdot t\right)\right] \end{array}$$

(4) $$\begin{array}{r} y\left(t\right)=\;A\cdot \exp\left[-\exp\left(\frac{\mu \cdot \exp\left(1\right)}{A}\left(\lambda -t\right)+1\right)\right] \end{array}$$

The *exp* represents an exponential function such that exp(x) = e^x^, where e is Euler's number. *A* represents the amplitude or peak growth value in the given environment, which corresponds to stationary phase and the maximum carrying capacity; *r* is the intrinsic growth rate coefficient; *y*_0_ is the initial population size; μ is the maximum slope of the growth curve, and *t* is time. For equations in which the maximum specific growth rate (μ_max_) was not a fit parameter; it was calculated by taking the derivative of *y*(*t*) evaluated at a software-derived point of maximum growth using parameter estimates for A, *r*, and *y*_0_.

Parameter estimates were derived in R 3.6.1 (R Core Team; R Foundation for Statistical Computing, Vienna, Austria) using curve fitting functions of easynls, growthcurver, and grofit packages (Kahm et al., 2010; Sprouffske and Wagner, 2016; Arnhold, 2017). The best parameter values for each function were found via Levenberg-Marquardt non-linear least-squares algorithm (Moré, 1978). The levels of fit between software packages varied and provided slightly different parameter values for each dataset. Relative area under the curve values, however, remained consistent across assessments within the same fitted models (i.e., Gompertz and Gompertz with software “A”) and with direct calculation of Riemann sums of integral of curves. The area under the optical density-based growth curves was calculated via two methods for each data set. The fitted models were integrated from the time of infection (*t* = 0) to the time-point delineating the end of the exponential growth phase of the uninfected host growth curve (i.e., the control). This process was used for each data set resulting in successful fits to Equations (1)–(4) (above) as well as other growth models (not shown here). The second method for calculating AUC values was through Riemann sums, also known as the trapezoidal approximation method (Hasenbrink et al., 2005). Equation (5) shows the formula utilized for calculating the AUC with this method.

(5) $$\begin{array}{r} AUC_{\text{Riemann}}=\;\overset{n-1}{\underset{i=1}{\sum }}\left(\frac{y_{i}\;+\;y_{i+1}}{2}\;\times \;(t_{i+1}-\;t_{i}\right)) \end{array}$$

Area under the curve values for virally challenged hosts were then standardized to the AUC value for the averaged uninfected host control growth curve in order to calculate the percent inhibition (PI) for the individual growth curves (see Tables S1–S3). The equation used to calculate the percent inhibition of growth is shown in Equation (6) below, from Rajnovic et al. (2019) which is reformatted in Equation (7). For a full discussion of this method (see Ceballos and Stacy, under review).

(6) $$\begin{array}{r} PI=\;\frac{AUC_{control}\;-\;AUC_{\inf}}{AUC_{control}} \end{array}$$

(7) $$\begin{array}{r} PI=\;1-\;\frac{\;AUC_{\inf}}{AUC_{control}} \end{array}$$

The left and right bounds of the integration are key parameters affecting the area under the curve metric. For these viral growth curves, the point of first difference is at time *t* = 0 when virus is added. The upper bound of the growth curve was originally chosen to be the intercept of the line produced by the maximum growth rate and the fitted asymptotic value. This method was not used because it does not include the transition period between the exponential growth phase and the stationary phase of growth, an area of the growth curve which we considered to be worthy of inclusion. Datapoints at and beyond stationary phase were excluded from AUC analysis because they disproportionately value the asymptotic (A) parameter, which undermines the robustness of the AUC value as a unifying metric of growth curve changes.

Virus replication data for SSV8 were fit to an exponential growth model both based on peak values and AUC calculations (with similar patterns emerging with either approach):

(8) $$\begin{array}{r} y\left(t\right)=Ae^{\left(t/\tau \right)} \end{array}$$

Parameter A is the initial population prior to infection and 1/τ is the growth rate constant.

For SSV9-infected cultures, average OD-based growth was fit to a sinusoidal function:

(9) $$\begin{array}{r} y\left(t\right)=Ae^{\left(-t/\tau \right)}\;\cdot \sin\left(\omega t\right) \end{array}$$

Term Ae^−t/τ^ represents the exponential decay of the oscillation amplitude whereby A is the driving amplitude and 1/τ is a decay rate constant. In the second term, ω is the frequency variable. The average growth curve of SSV9-infected cultures was fit to a damped sinusoidal function simply because this function best described the cyclical bursts in growth that had increasing smaller amplitude and which were each followed by rapid declines in host population (as determined by optical density readings). However, for SSV9 replication, a Gaussian function was used to fit the dataset.

(10) $$\begin{array}{r} y\left(t\right)=Ae^{-\frac{{\left(t-t_{c}\right)}^{2}}{{2w}^{2}}} \end{array}$$

Parameter A represents the amplitude of the peak function; *t*_*c*_ is the time at which the center of the peak occurs; and, w represents the width of the peak function at one-half the peak amplitude. The “goodness-of-fit” of these data sets (assessed by adjusted *r*^2^ values) to the sinusoidal and Gaussian, respectively, prompted their use. For the sinusoidal fit, the adjusted *r*^2^ value was 0.90 (see Figure S2B). For the Gaussian fit to the SSV9 virus replication data, adjusted *r*^2^ = 0.94. Note that *r*^2^ values typically were between 0.90 and 0.99, except for fits to the SSV9-infected host growth curves, which failed or had *r*^2^ < 0.25.

### qPCR for Determination of SSV9 Genome Copy Number

SYBR green-based qPCR was used to quantify the number of SSV9 genomes for inocula and end-point infection. Two PCR primers were employed: SSV9F (5′-GTGAAGCGACCAACATAGGTGCAA-3′) and SSV9R (5′-GTTGCGTTTGTACCGGTTACGCTA-3′)—targeting the single-copy gene *vp1*, which encodes a major SSV structural protein (Bautista et al., 2015). Standard curves were generated with 10-fold serial dilutions (10^8^–10^0^ copies) of the *vp1* gene fragment (138-bp) cloned into a TOPO TA pCR2.1 plasmid (Invitrogen). Copy number in each standard was calculated using formula:

(11) $$\begin{array}{r} gene\;copy\;number\;=\\ \;\frac{\left[sample\;concentration\;\left(\frac{ng}{\mu l}\right)\right]{\;\cdot \;N}_{A}\;}{\left[fragment\;length\;\left(bp\right)\right]\;\cdot \;1x10^{9}\;\left(\frac{ng}{g}\right)\;\cdot \;660\;\left(g/mol\right)\;}.\; \end{array}$$

where, 660 g/mol is the molecular weight of one base pair, *N*_*A*_ is Avogadro's number, and 1 × 10^9^ is used to convert units to nanograms. Each qPCR reaction was performed on a Rotor-Gene Q (Qiagen, Inc.; Maryland, USA) and consisted of 1× Green/ROX qPCR Master Mix (Fermentas, Inc.; Ontario, Canada), 3 pmol of each primer, 1 uL of DNA template, and nuclease-free water to a final volume of 20 mL. Three technical replicates were performed of each standard and samples. The qPCR cycle parameters were as follows: 98°C for 2 min, followed by 40 cycles of 98°C for 5 s and 20 s at 60°C. A melt curve analysis was performed after each run from 65 to 95°C in 0.5°C increments at 2 s intervals, to ensure specific amplification of a single target, and no primer dimer formation. The qPCR amplicons were resolved in 2% agarose gels to assess amplicon sizes for target-specific amplification. Reaction mixtures with sterile water (no-template DNA) or pCR2.1 DNA without insert served as a negative controls to control for false positives. Assays showed amplification efficiencies of 100% ± 10% (i.e., with a slope between −3.6 and −3.1), consistency across replicate reactions, and linear standard curves (*R*^2^ > 0.970). The absolute quantification method was used to calculate the number of viral genomes per mL of culture.

## Results

### Virus Yields and Confirmation of SSV Infection

To ensure that virus infection was a cause of host growth decline, presence of virus was determined by one (or more) of four distinct methods: electrospray ionization/mass spectrometry (ESI/MS) in units of virus particles per milliliter (VP mL^−1^); serial dilution plaque-like assays in units of halo-forming units per milliliter (hfu mL^−1^); virus-like particle (VLP) counts using transmission electron micrographs (VLP mL^−1^); and/or, quantitative polymerase chain reaction (qPCR) in terms of the total number of viral genomes per milliliter (v_g_ mL^−1^). For infection assays, end-point titers were also determined by one or more of these methods. ESI/MS and plate assays provided the most consistent results.

**Figure 1:**
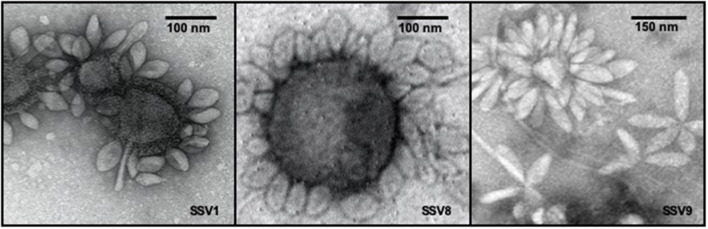
Transmission electron micrographs of SSV particles. Transmission Electron Microscopy (TEM) demonstrates the presence of spindle-shaped virus particles after harvest and concentration steps at end-point of infection assays as well as during the preparation of virus stocks for liquid culture infection assays and halo assays. TEM images of SSV1 **(left)**, SSV8 **(middle)**, and SSV9 **(right)**.

Growing 600 mL liquid cultures of permissive host, each infected with a single SSV strain, to late *log phase* growth (e.g., OD_600nm_ = 0.8 = 5.4 × 10^8^ cells mL^−1^) resulted in comparable virion yields as shown by electrospray ionization/mass spectrometry (ESI/MS) spectra (Figure S1). For this culture volume, SSV8 and SSV9 suspensions result in ~2 × 10^8^ to 4 × 10^8^ VP mL^−1^. SSV1 yields are typically 50–80% lower. Thus, 1.2–2.0 L of SSV1-infected culture were required to ensure stocks were the same order of magnitude and comparable to SSV8 and SSV9. SSV suspensions were then diluted to produce working stocks with equivalent titers (e.g., ~1 × 10^8^ VP mL^−1^). Verification of the presence of SSV virions in virus suspensions was accomplished by transmission electron microscopy (Figure 1). Electron micrographs typically feature SSV particles either in *rosettes*, where multiple virons are attached at one end presumably via electrostatic and hydrophobic interactions (Quemin et al., 2015) between tail fibers (see Figure 1, right panel), or to cell debris (e.g., membrane remnants) by their tail fibers (see Figure 1, left and middle panels). Depending upon the host in which the SSVs are cultivated, slight changes in morphology (i.e., elongation) may present. However, SSV9 consistently shows a more elongated virion (Figure 1, right panel).

**Figure 2:**
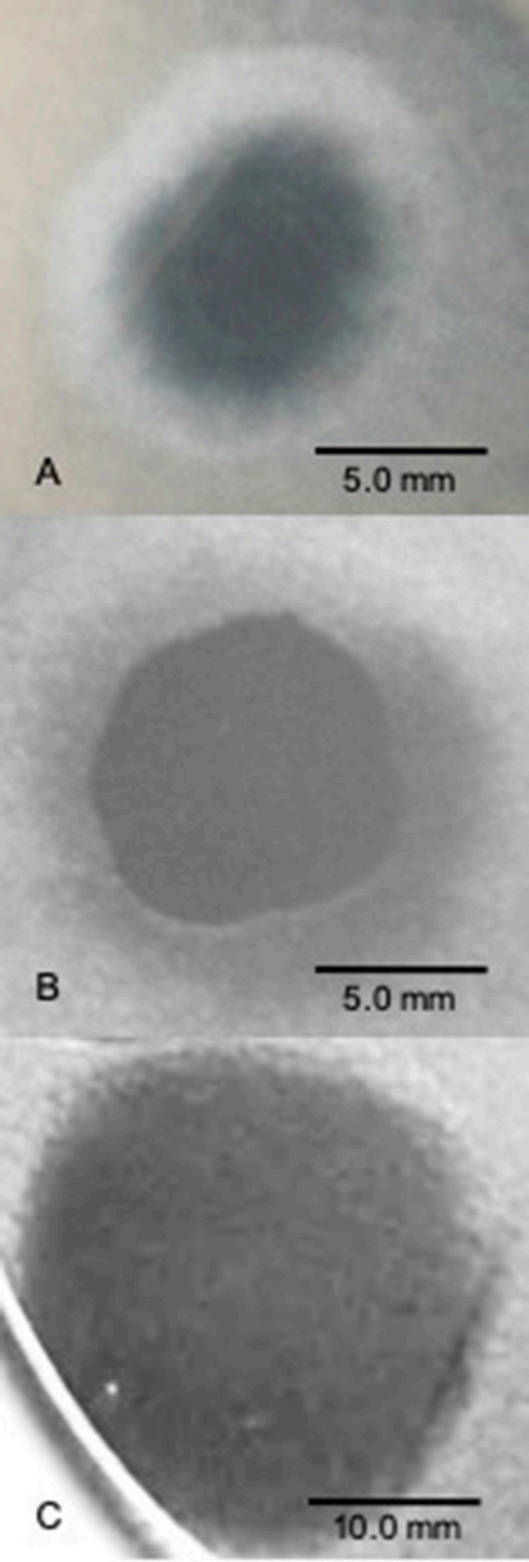
SSV spot-on-lawn halo assays. A 2 μL drop of virus suspension was spotted on lawns of *Sulfolobus solfataricus* strain Gθ to determine whether a viral infection can be established and the nature of the virus-host interaction in terms of halo “phenotype” (size, shape, boundary type). **(A)** SSV1 forms broad diffuse, turbid halo. **(B)** SSV8 (a.k.a., SSVRH) makes a broad diffuse, turbid halo with a sharper boundary between the direct application point and halo leading edge; **(C)** SSV9 (a.k.a., SSVK1) yields a large and complete clearing of host lawn.

**Figure 3:**
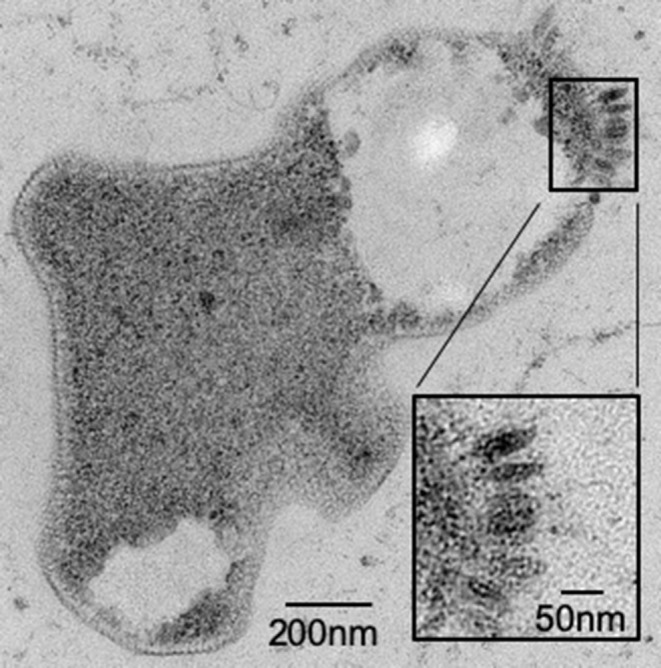
*Sulfolobus* strain Gθ infection by SSV verified by TEM. Transmission Electron Micrograph (TEM) of a 30–60 μm epoxy-impregnated section (60,000X magnification) from a host cell infected with SSV8. A closer view (inset) of this micrograph reveals fusiform or “spindle-shaped” virus-like particles on the surface of the cell membrane.

It has been previously reported that not all species within the family *Sulfolobaceae* are susceptible to SSV infection (Ceballos et al., 2012). Thus, to ensure that there was active infection in the virus-host pairings selected for this study, two validation steps were taken. First, *spot-on-lawn* plate assays were used as a qualitative (i.e., yes or no) verification that a given SSV strain is able to infect a specific strain of the family *Sulfolobaceae* (Figure 2). Second, small-scale test infections in liquid culture were performed and TEM images were obtained (Figure 3).

To characterize halo phenotype and validate SSV infectivity, 2 μL of virus suspension was used in *spot-on-lawn* plate assays. On multiple susceptible hosts (see Ceballos et al., 2012), SSV1 consistently induces a smaller more diffuse halo phenotype (Figure 2A). The halo phenotypes induced by SSV2 and SSV3 vary depending upon the host (data not shown) and are less consistent than those produced by SSV1 and SSV8. SSV8 consistently produces large halos on hosts with diameters comparable to those produced by SSV9. However, SSV8 halos have diffuse boundaries (Figure 2B); whereas SSV9 boundaries are sharp (Figure 2C). To control for sulfolobicin-induced halos (false positive SSV infection), negative controls using ultrafiltrate (10 kDa MWCO) were also spotted on lawns as a negative control. Sulfolobicins are reported to be ~20 kDa (Prangishvili et al., 2000).

Together spot-on-lawn plate assays and electron micrographs from liquid culture infections validate whether a specific SSV strain is able to productively infect a putative host strain. Productive SSV1, SSV2, SSV3, SSV8, SSV9, and SSV10 infection on host strain Gθ was confirmed through these procedures prior to conducting large-scale liquid culture assays. For large-scale trials, we used only SSV1, SSV8, and SSV9 because of consistency in halo phenotypes and due to the fact that SSV8 exhibits one of the highest levels of relative virulence (and, thus is a comparator for SSV9) across multiple susceptible hosts.

### Host-Growth After Infection by Various SSVs

**Figure 4:**
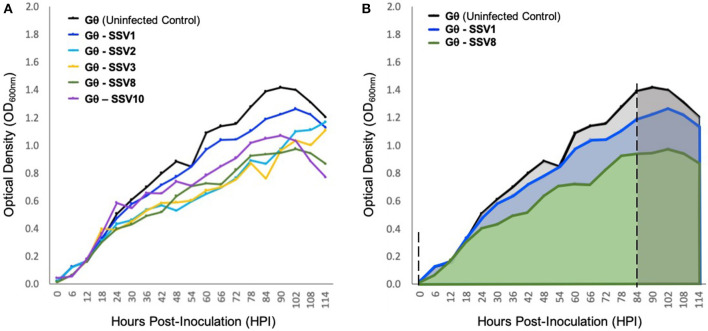
Growth of host strain *Sulfolobus* Gθ during SSV infection. **(A)** *Sulfolobus* strain Gθ infected with SSV1, SSV2, SSV3, SSV8 (a.k.a., SSVRH), and SSV10 (a.k.a., SSVL1) at a MOI = 0.01. **(B)** Graphical representation of percent inhibition of *Sulfolobus* strain Gθ via area-under-the-curve (AUC). All growth curves fit with high coefficients of determination (*R*^2^ values) to Gompertz Models or Logistic Growth Models (see Table S1). The asymptotes (dashed) indicate point of inoculation until end of the exponential growth phase/onset of stationary phase.

Liquid culture assays using SSV1, SSV2, SSV3, SSV8, and SSV10 infecting the universally susceptible host strain *Sulfolobus* strain Gθ (Cannio et al., 1998), reveal that host growth (μ_max_) is slowed and the peak cell density upon entering the *stationary phase* (i.e., *N*_*asymptote*_) is reduced compared to uninfected control (Figure 4). Individual (i.e., single virus-single host) infections with SSV1, SSV8, or SSV10 on strain Gθ show archetypal host growth with these reportedly non-lytic SSVs. Infection with either of two SSVs isolated from two geographically-distinct Icelandic geothermal regions—SSV2 (from Reykjanes) and SSV3 (from Krisovic)—also show reductions in μ_max_ and area-under-the curve (AUC) when compared to controls. Despite SSV2 and SSV3 infections exhibiting slightly different host growth profiles (Figure 4A, cyan and mustard lines) due to slower growth to stationary phase, data still fit a Gompertz model (Gompertz, 1825; Laird, 1965) with high coefficient of determination values (Table S1). Measures of percent inhibition were derived by AUC calculations (Zwietering et al., 1990) for the infected host growth and dividing by the AUC for the uninfected control (Figure 4B). Using this quotient, an index of relative virulence (*V*_*R*_) between strains on a given host was generated.

Using a Gompertz model and AUC as a measure of percent inhibition (PI), SSV1 exhibits the lower PI (8.57%) while SSV8 has a significantly higher PI (28.44%) with R^2^ values for the Gompertz of 0.9875 and 0.9775, respectively. Employing Riemann Sums or alternative models (i.e., Logistic Growth) does not significantly alter relative virulence (*V*_*R*_) for SSV1 and SSV8 (see Table S1). Regardless of analytical approach, the general trend in virulence of SSVs on *Sulfolobus* strain Gθ is: SSV1 < SSV10 << SSV2 ≈ SSV3 < SSV8. Note that AUC measures suggest that SSV2 and SSV3 have a slightly greater PI than SSV8. However, note that AUC for SSV2 and SSV3 is underestimated due to a lag in both strains reaching stationary phase compared to the other SSVs (Ceballos and Stacy, under review). Therefore, in subsequent trials, SSV1 and SSV8 were chosen as comparators for SSV9.

### Host-Growth After Infection With SSV9

**Figure 5:**
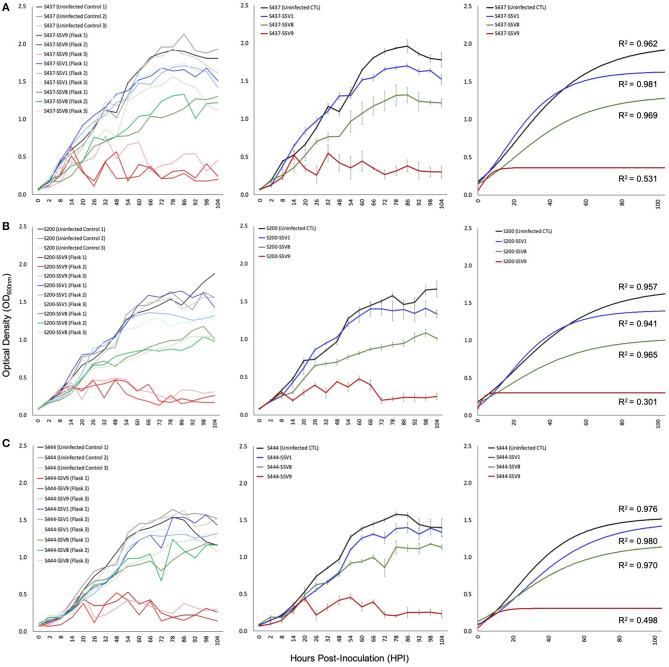
Growth profiles for susceptible allopatric host strains infected with SSVs. Under equivalent Multiplicity of Infection (MOI = 0.1), allopatric hosts show classic Gompertzian growth when infected with either SSV1 or SSV8. However, cultures of the same hosts infected with SSV9 exhibit drastic growth inhibition. Growth in SSV9-infected cultures are “saw-toothed” with damped oscillations. Growth curves from liquid culture infection assays using SSV1, SSV8, and SSV9 on susceptible allopatric host strains: **(A)** S437 (DSM1617 from the Deutsche Sammlung von Mikroorganismen und Zellculturen), a type strain (a.k.a., *S. solfataricus* P2) isolated from a hot spring near Pisciarelli, Italy; **(B)** S200 (a.k.a., *S. icelandicus* HVE 10/4 from the Hveragerdi thermal region of Iceland); and, **(C)** S444 (an isolate derived from Lassen Volcanic National Park, California, USA).

Unlike infection with SSV1, SSV2, SSV3, SSV8, or SSV10, *Sulfolobus* strain Gθ infected with SSV9 did not grow with Gompertzian-like (Gompertz, 1825; Laird, 1965) dynamics. In contrast, rapid and significant inhibition of culture growth was observed in cultures infected with SSV9. This pattern was observed for several Sulfolobales strains over multiple replicates (3× per trial) with each strain as shown in Figure 5 and multiple trials (2–4×) for each virus-host pairing. Furthermore, all susceptible Sulfolobales strains tested serve as allopatric hosts to SSV9.

Infection assays using SSV1 (Beppu, Japan), SSV8 (Yellowstone National Park, USA), and SSV9 (Valley of Geysers, Kamchatka, Russia) were used to infect susceptible hosts: S200 (a.k.a., *S. icelandicus* HVE 10/4 from the Hveragerdi thermal region of Iceland); S444 (an isolate derived from Lassen Volcanic National Park, California in the USA); and, S437 (a.k.a, DSM1617; isolated from a hot spring near Pisciarelli, Italy). With the exception of SSV1 infection of strain HVE 10/4, all host growth curves show significantly reduced *N*_*asymp*_, AUC, and/or μ_max_ for SSV1 and SSV8 when compared to uninfected controls. However, when each host was infected with SSV9, a distinct host growth profile emerged. Specifically, Gompertzian Models failed to adequately represent the SSV9 infection data. Instead, non-Gompertzian cyclical spike patterns are consistently observed in host growth. Low *R*^2^ values (<0.5) emerged for both individual traces and averaged growth curves for SSV9 (Figure 5, red traces), whereas, high *R*^2^ values (>0.9) were typical for all other SSVs infecting these same hosts (Table S2).

### Host-Growth Profile of SSV9 Is Not MOI-Dependent

**Figure 6:**
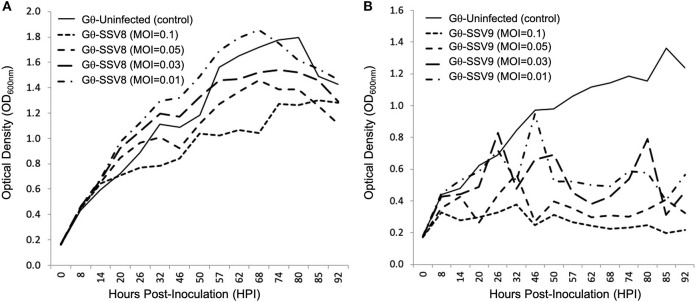
Growth of *Sulfolobus* strain Gθ infected at Different MOIs: SSV8 versus SSV9. **(A)** Under different Multiplicity of Infections (0.1, 0.05, 0.03, and 0.01), strain Gθ infected with SSV8 retains Gompertzian growth. **(B)** Host strain G infected with SSV9 using the same MOIs consistently results in the cyclical “saw-toothed” profile characteristic of SSV9 infection (and typical of lytic virus systems).

To determine if the unusual growth profiles of SSV9-infected hosts are MOI dependent, dilutions of SSV8 and SSV9 stocks were used in liquid culture assays on host strain Gθ (Figure 6).

The typical host growth pattern shown for SSV infection (i.e., Gompertzian-like growth) is observed for all SSV8 infections on *Sulfolobus* strain Gθ regardless of MOI (Figure 6A). A positive correlation between SSV8 MOI and percent inhibition of host growth was generally observed. However, for the lowest viral stock dilution (MOI = 0.01), the PI was not significantly different from uninfected control despite productive infection. The atypical host growth profile for host infected with SSV9 also persisted (Figure 6B). Amplitudes of host growth spikes change with an inverse relationship in response to MOI; however, there was no switch to the Gompertzian-like effect even at the lowest SSV9 MOI.

### End-Point Infection Assays Indicate SSV9 Lytic Replication

**Figure 7:**
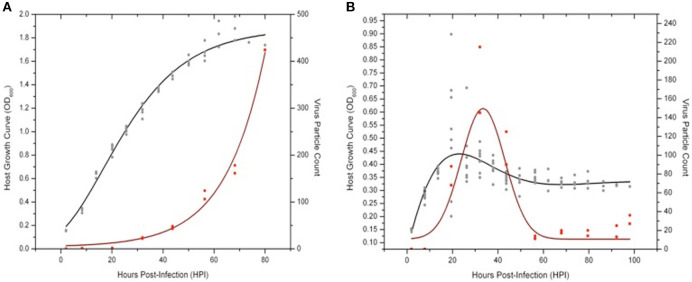
Virus-host dynamics: SSV8 vs. SSV9 infections in *Sulfolobus* strain Gθ. Using a MOI = 1 to ensure virus particle detection via ESI/MS, host strain Gθ was infected with SSV8 or SSV9. **(A)** *Sulfolobus* strain Gθ at an OD_600_ = 0.15 − 0.20 is infected with SSV8 at MOI = 1 resulting in standard Gomperz-like culture growth with a concomitant increase in virion count over time. **(B)** *Sulfolobus* strain Gθ at OD_600_ ≈ 0.15 − 0.20 is infected with SSV9 at MOI = 1 resulting in an average peak density of OD_600_ ≈ 0.45 followed by a decrease in cell density and a sudden increase in SSV9 particle count, peaking at 32 HPI. Cell density stabilizes at OD_600_ ≈ 0.30 − 0.35 from 60–90 HPI. SSV9 particle count drops to the detectable limits of the ESI/MS (~1 × 10^6^ − 2 × 10^6^ virus particles/mL).

To investigate both host and virus population dynamics in liquid culture infection assays, two large-scale trials (i.e., 14–20 replicates) at higher observation frequency (every 4 h) were conducted with *Sulfolobus* strain Gθ infected with either SSV8 or SSV9 at MOI = 1 (Figure 7). Two flasks were harvested every 12 h from the replicates to determine virus particle counts via ESI/MS. Sulfolobus strain Gθ infected with SSV8 showed typical Gompertzian-like host growth with a concomitant increase in virus particle count (Figure 7A) through 78 h post-infection (HPI).

SSV9-infected Sulfolobus strain Gθ showed a distinct pattern from the Gθ-SSV8 infection. SSV9-infected growth peaked (18 HPI) followed by a rapid drop in cell density (Figure 7B). The decrease in host density was followed by an increase in SSV9 particle count (32 HPI). Interestingly, other than the initial peak in host cell density, no noticeable subsequent peaks (dampened or accentuated) were observed (Figure 7B) as in prior experiments (see Figure 5).

It was also noted that the growth inhibition did not result in an optical density value of zero. Instead, cell density stabilized at approximately one-third the average peak value. Furthermore, SSV9 titer (via ESI/MS) decreased substantially (see Figure 7B, 60–100 HPI). Although the general growth profiles were consistent with prior trials for non-lytic vs. lytic release, these latter two features were not expected (see Discussion). Together, these data indicate that SSV8 pursues canonical non-lytic virion release expected of SSVs, while SSV9 lyses host strain Gθ. This is also supported qualitatively by the presence of significantly more cell debris in SSV9-infected cells in small-scale infections (Figure S2).

### SSV9 Lytic Replication May Be Limited to Allopatric Hosts

To determine if SSV9 lytic replication was dependent upon host strain allopatry, two isolates of Sulfolobales derived from volcanic hot springs in Kamchatka, Russia were also tested as plausible hosts for SSV9.

**Figure 8:**
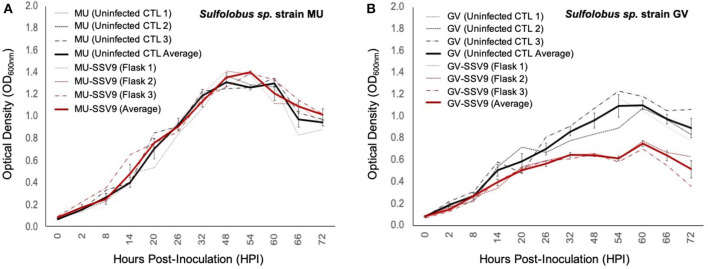
Impacts of SSV9 challenge on isolates from geothermal regions in Kamchatka. Isolates of Sulfolobales from geothermal regions of the Kamchatka peninsula were infected with SSV9. *Sulfolobus* sp. strain GV was derived from the same hot spring region as the host from which SSV9 was derived. *Sulfolobus* sp. strain GV is considered a sympatric host. *Sulfolobus* sp. strain MU was also derived from the Kamchatka peninsula but from a geothermal region approximately 256 km to the southwest of Geyser Valley. *Sulfolobus* sp. strain MU is quasi-sympatric to *Sulfolobus* sp. strain GV. **(A)** *Sulfolobus* sp. strain MU growth curves for SSV9-infected cultures exhibit no significant changes in μ_max_ compared to the uninfected control curves. **(B)** *Sulfolobus* sp. strain GV host growth curves for SSV9-infected cultures resemble canonical non-lytic replication.

Liquid culture infection assays confirm that strain MU is resistant to infection (Figure 8A) and that strain GV is susceptible to SSV9. Post-infection titers for strain MU infection trials did not show any detectable virus; while those for strain GV showed virus particle counts on the order of 10^9^ VP mL^−1^ indicating productive infection (Figure S4). The most striking result was that strain GV growth during SSV9 infection resembles the non-lytic archetype infection dynamics observed with other SSVs (Figure 8B).

AUC analysis for MU-SSV9 trials results in no significant growth inhibition (PI < 0.05), while the GV-SSV9 trial shows a significant PI (>0.20) with a convincing Gompertz fit (*R*^2^ = 0.934), indicating that SSV9 exhibits a non-lytic replication profile on a sympatric host, *Sulfolobus* sp. strain GV (see Table S3).

## Discussion

Previously published work demonstrated that some Sulfolobales are completely resistant to infection by well-characterized SSVs (i.e., SSV1, SSV2, SSV3, SSV8, SSV9, SSV10) while others are susceptible to subset or all of these SSVs in *spot-on-lawn* “halo” assays (Ceballos et al., 2012). Large plaque-like halos were observed on host lawns infected with SSV9 (using the same amount of virus) suggesting that SSV9 may be more virulent. However, the large clearings on host lawns generated by SSV9 appeared to be true plaques rather than the turbid halos characteristic of SSV infection. This raised the possibility that SSV9 is lysing its host rather than using non-lytic budding as a means of virion release. Although the halo assay is sufficient for determining whether a given host is susceptible (or not) to a specific SSV, it offers limited information regarding the dynamics of infection. In the present study, liquid culture assays and host growth curve analyses were used to test the hypothesis that *SSV9 employs a lytic replication strategy rather than the canonical non-lytic replication (i.e., release via virus budding) characteristic of other SSVs*.

### SSV Infection in Liquid Culture

Growth curve analysis provides insights into the nature of virus-host infection dynamics that are not resolvable through plate-based halo assays. Using host growth curve analyses, it is not only possible to determine percent inhibition and relative virulence of a different SSVs on a host (see Figure 4A) but it is also possible to gain insights into rates of virus replication/transmission as well as virion release strategy. By measuring titers of inocula at the beginning of an infection trial, establishing equivalent MOI for different virus-host pairings, and taking end-point titers, a quantitative assessment of how many virus particles are present in the culture at the time of inoculation vs. how many progeny virions are present at the end-point was possible. However, this method of comparing pre-infection titer and post-infection titers may have been confounded by differential stability of distinct SSV strains in the high temperature (76–80°C) and low pH (3.0–3.4) culture environment. Although SSV8 and SSV9 appeared to be more resilient in solution, SSV1 did not seem to be as stable (Figure S3). Thus, viral fecundity may have been underestimated by virus count since some virus particles (e.g., SSV1 virions) breakdown during the course of the infection assay. Still, end-point virus count is needed to confirm productive infection. Specifically, if the number of virus particles at end-point exceeds the number of virus particles used for inoculation, then this indicates that the infection was productive. However, if virus count at end-point is equal to or less than virus particle count at inoculation, then it can be argued that no infection occurred.

Although it can be argued that a highly unstable virus strain could rapidly breakdown yielding a false negative for infection, TEM (see Figures 1, 3) and spot-on-lawn halo assays (see Figure 2) are used as secondary methods to validate host susceptibility/SSV strain infectivity.

### Archetypal Non-lytic SSV Replication and Host Growth Profiles

Despite limitations in determining end-point virus count due to differences in virion stability between the different SSVs, liquid culture assays permit a quantitative assessment of relative virulence (*V*_*R*_) by calculating percent inhibition (PI) of each SSV on a given host strain (see Figure 4B). The archetypal non-lytic replication strategy of SSVs allows quantitative assessment of *V*_*R*_ based on comparing *N*_*asymptote*_, μ_max_, and/or AUC in SSV-infected vs. uninfected controls.

### Atypical Infection Dynamics of SSV9 on Allopatric Host Growth

Archetypal SSV infection (see Figure 4) induces Gompertz-like host growth profiles (Ceballos and Stacy, under review), indicative of non-lytic infection, which is consistent with turbid halo, instead of plaque, formation on spot-on-lawn plate assays. However, when SSV9 is used to infect a series of susceptible allopatric hosts, atypical infection dynamics emerge in the host growth curve. Specifically, a deep inhibition, which manifests as “saw-tooth” or “serial spike” profile in the host growth curve, occurs suggesting a different replication process (Figure 5, red traces). Although it may be suggested that SSV9 is simply more virulent on the susceptible host, this argument is refuted by the fact that neither a Gompertz or Logistics growth models fit reasonably to the SSV9-infection host growth data (as indicated by very low R^2^ values).

### Atypical Infection Dynamics of SSV9 Are Not MOI Dependent

Further support for unique SSV9 infection dynamics rests in the inability to elicit the archetypal non-lytic growth profile by manipulating MOI (see Figure 6B). Even under low MOI, SSV9 continues to induce a cyclic spike profile in the host growth curve. Likewise, varying MOI for SSV8 does not change the archetypal non-lytic growth profile to one resembling SSV9 infection. To determine if a change in the SSV8-infect host growth profile could be forced by a concentrated inoculum, a 20:1 virus concentrate was used (MOI = 2). Although the culture exhibited a strong depression in growth *(not shown)*, the curve still fit a Gompertz Model with high coefficient of determination (*R*^2^ = 0.87) and did not show a cyclic growth profile. Furthermore, virus particle count at the end of a 20:1 viral concentrate trial (not shown) was 2-fold greater than the initial inoculum, indicating that the depression in the growth curve was not due to lysis-from-without (Delbrück, 1940) but due to productive infection.

### SSV Population Dynamics Support Lytic Replication

Larger-scale liquid culture assay trials (with a large number of replicate flasks) allow replicates to be harvested periodically during the course of infection to monitor changes in virus titer in parallel to host growth (Figure S2). Sampling at shorter intervals (i.e., every 4 h), infection with SSV8 (a non-lytic replicator) shows an increase in virus particle count concomitant with host growth. However, SSV9 infection of the same host strain (i.e., Gθ) shows sharp peak in virus particle count ~12 h after a peak and a notable rapid drop in host cell density. This is consistent with lytic virus release or a “burst” (Figure 7B). Interestingly, even though the assay continued to ~98 HPI, no subsequent peak-and-crash of host (or virus) occurred after the first cycle (~30 HPI). Moreover, optical density (a proxy for host cell density) remained steady at OD_600nm_ ≈ 0.3 from 60 to 98 HPI. The reason underlying the stability of host cells density with no additional SSV9-induced lysing or SSV9 virion production is not clear. There is one report suggesting that SSV9 challenge may induce dormancy in viable hosts (Bautista et al., 2015), which may be the reason for sustained absorbance at OD_600nm_ ≈ 0.3. Indeed, dormant cells would not likely support virus production and may render cells resistant to infection.

Another report suggests that group II chaperonin complexes may form highly stable networks of chaperonin complex filaments at the intracellular surface of membrane (Trent et al., 2003). Cell membrane-associated chaperonin complexes as filaments or two-dimensional arrays can maintain cell shape even if cells are not viable. Under scanning electron microscopy (SEM), cells from infected culture, which are likely to be non-viable “ghost cells” due to the presence of large holes in the cell membrane, maintain a round lobed three-dimensional structure that could absorb 600 nm light (*also reported in* Quemin et al., 2016). Whether the absence of subsequent cycles of host recovery, SSV9 infection, and lytic bursting beyond the initial cycle are due to cells becoming dormant or whether these are “ghost” cells requires further study. Nonetheless, host growth curves for SSV9-infected strains display atypical profiles that do not fit to Gompertzian (or Logistic Growth Models) as is expected for non-lytic virion release but rather represent lytic bursts of virion release. Thus, both spot-on-lawn halo assays and liquid culture assays suggest that SSV9 lyses host. Whether this is classical lytic replication (as seen in bacteriophage) or if lysis is a result of virion activity at the membrane, which induces membrane breakdown, remains uncertain. It is possible that a “lysis-from-within” phenomena is responsible whereby aggressive virus egress is inducing gross cell lysis in allopatric hosts, which have limited coevolutionary history with SSV9; whereas the sympatric host is adapted to support typical SSV budding. Whether SSV9 infection is truly lytic replication or a “lysis-from-within” phenomenon, is uncertain. Nonetheless, small-scale infection assays further support that cells are lysed. Specifically, infection with SSV9 generates visible cell debris at the same time point of infection compared to infection with other SSVs, which do not (see Figure S2).

### SSV9 Lytic Replication May Depend on Host Allopatry

Although SSV9 lytic behavior does not appear to be dependent upon MOI and seems to be an inherent property of the SSV9 replication strategy, all susceptible hosts initially tested were *allopatric* hosts isolated from geothermal hot springs thousands of kilometers away from where SSV9 was isolated. Yet, when two Sulfolobales—one from the same geothermal region from which the original SSV9 was isolated and another from a distant spring (ca. 250 km) within the same area (i.e., Kamchatka, Russia)—were challenged with SSV9, unanticipated dynamics emerged.

The *quasi*-sympatric strain MU was not susceptible to SSV9 in spot-on-lawn assays. Moreover, in liquid culture assays with strain MU, there were no significant differences in *N*_*asymptote*_, μ_max_, or AUC between the SSV9-infected and MU uninfected controls (Figure 8A). This is not surprising since other Sulfolobales are reported to be resistant to SSV infection (Ceballos et al., 2012). Yet, when SSV9 was used to challenge the *sympatric* strain GV, there were unexpected results. Specifically, it appears that SSV9 not only infects strain GV but infection follows canonical non-lytic replication characteristic of other SSVs (Figure 8B). It is possible that coevolution of host and virus has resulted in a host cell membrane that is more resilient to the sympatric virus assembly mechanisms at the membrane surface, while membranes of allopatric hosts are more sensitive to SSV9 assembly resulting in cell lysis.

Recently, a series of reports (Sakai and Kurosawa, 2018; Tsuboi et al., 2018) proposed a re-organization of *Sulfolobaceae* into four distinct genera: *Sulfolobus* (Brock et al., 1972), *Stygiolobus* (Segerer et al., 1991), *Sulfurisphaera* (Kurosawa et al., 1998), *Saccharolobus* (Sakai and Kurosawa, 2018; Tsuboi et al., 2018)—with the latter being the most recently defined based largely on phylogenetic distance.

Whether SSV infectivity and relative virulence is correlated with these newly defined genera remains to be determined. In terms of our prior work (Ceballos et al., 2012) and this current study, this reorganization means that SSVs infect select strains belonging in at least two of these genera (i.e., *Saccharolobus* and *Sulfurisphaera*). The full impact of this taxonomic reorganization, especially with regard to biogeography and physiological differences is being assessed. Interestingly, *Sulfurisphaera tokodaii* (Tsuboi et al., 2018) and *Sulfurisphaera ohwakuensis* (Kurosawa et al., 1998) share a similar infection pattern to *Sulfolobus* sp. strains MU and GV. Specifically, *S. tokadaii* appears to be resistant to SSV infection, like strain MU; while, *S. ohwakuensis* is susceptible to at least a subset of SSVs tested (Ceballos et al., 2012), like strain GV. Although re-organization of the phylogenetic tree for the family *Sulfolobaceae* requires the community to revisit and reassess previously published work, the non-lytic growth profiles observed in SSV9 infections raises a question of whether sympatric vs. allopatric virus-host evolution or phylogenetic relatedness or both determines replication strategy. Since lytic replication typically results in greater virulence (than non-lytic replication), there is also the question of whether a switch in replication strategy over evolutionary timescales (i.e., lytic to non-lytic) is part of a more generalizable pattern of attenuation in the virus-host coevolutionary “arms race” (van Valen, 1973; Dawkins and Krebs, 1979). Furthermore, the genetic substrates underlying the emergence of distinct replication strategies in SSV systems remain unknown. Employing high throughput sequencing methods (e.g., nanopore sequencing) and advanced analytical techniques may resolve the impacts of specific genetic factors on virus-host dynamics and quantify relative virulence between non-lytic vs. lytic phenotypes, respectively (Ceballos and Stacy, under review), may help to address the aforementioned questions and identify genetic elements that predispose viruses to lytic vs. non-lytic replication strategy.

## Data Availability Statement

All datasets generated for this study are included in the article/Supplementary Material.

## Author Contributions

RC and KS conceptualized the study. RC performed the liquid culture and plate assays and did the virus titering using ESI/MS with instrumentation at BVS, Inc. (Hamilton, MT). CD performed the plate assays and conducted the SSV thermal stability and adsorption studies, and also did the virus titering using serial dilution plaque assays. CS and RC fit the data to mathematical models and determined N_asymptote_, μ_max_, and AUC for all liquid culture assays. RC and CD produced the TEM and SEM images. EP-C performed the qPCR-based virus titers. RC prepared the initial drafts of the full manuscript and incorporated the final revisions. KS contributed to the sections of the writing and revised iterations of the manuscript.

## Conflict of Interest

The authors declare that the research was conducted in the absence of any commercial or financial relationships that could be construed as a potential conflict of interest.

## Supplementary Material

Figure S1 SSV titers via electrospray ionization mass spectrometry (ESI/MS). Concentrated viral suspension is diluted 1:10 and mixed with ionization solution (see section Methods). SSV particle count in 120 nL of suspension is monitored by a particle detector for 3 min. The area-under-the-curve (AUC) provides an estimate of titers. Each SSV has a characteristic spectrum. SSV1 (blue) is generally a single broad peak. SSV8 (green) exhibits a large single peak with a shoulder. SSV9 (red) typically presents as a double-peak. Since ESI/MS measures are based on mass:charge ratios of spherical particles and SSVs are fusiform (quasi-elliptical) shoulders and double peaks may be due to different orientations between aerosolization and triggering the detector. Alternatively, morphologically different populations may be present, including a sub-population of defective virus-like particles such as those that have lost their tails, which generate a shoulder or second peak.

Figure S2 Cellular Debris in SSV9-infected but not in SSV1-infected Small-scaled Cultures. **(A)** SSV9-infected *Sulfolobus* strain Gθ has cell debris visible at 22 HPI; whereas, **(B)** SSV1-infected Gθ at 22 HPI, and **(C)** uninfected Gθ uninfected control do not. Cultures were allowed to settle overnight. Both infections were conducted at an MOI = 3. Cell debris is another line of evidence suggesting that SSV9 induces host cell lysis.

Figure S3 SSV viability after exposure to culture environment. At 75°C (pH 3.2) SSV9 (red) retains viability for up to 25 h; while titers of SSV1 (blue) decrease rapidly after 6 h in the same high temperature acid conditions. Plate-based serial dilution plaque-like “halo” assays (hfu/mL) were used to determine titers for each time point for SSV9 and SSV1. SSV9 virus particles (red) have greater stability in the high temperature and acid environment.

Figure S4 SSV9 genome abundance via quantitative polymerase chain reaction (qPCR). Viral genome counts were measured via qPCR after infections of *Sulfolobus* sp. strains MU and GV with SSV9. Liquid cultures of *Sulfolobus* sp. strains MU and GV were infected with SSV9 at an MOI = 0.1 (dark grey bars) from a SSV stock titered at 1.03 × 10^10^ vg/mL (black bar) followed by incubation for 72 h at 78°C (90 RPM shaking). At 72 HPI, titers were determined by qPCR from culture supernatant. SSV9 genomes per mL in MU culture dropped five orders of magnitude to within the lowest detection limit (medium grey bar), which indicates no productive infection. SSV9 genomes in GV culture increased by two orders of magnitude (light grey bar), indicating that *Sulfolobus* sp. strain GV is permissive to SSV9 replication.

Table S1 Percent inhibition calculations for SSV-G infection: Figure 4.

Table S2 Percent inhibition for SSV infections on allopatric hosts: Figure 5.

Table S3 Percent inhibition for SSV9 challenges on sympatric sulfolobales: Figure 8.
