# Supplementary figures and images for: Host-Dependent Differences in Replication Strategy of the Sulfolobus Spindle-Shaped Virus Strain SSV9 (a.k.a., SSVK1): Infection Profiles in Hosts of the Family Sulfolobaceae
Source: Front Microbiol. 2020 Jul 14;11:1218. doi: 10.3389/fmicb.2020.01218 (PMC7372142; doi:10.3389/fmicb.2020.01218)

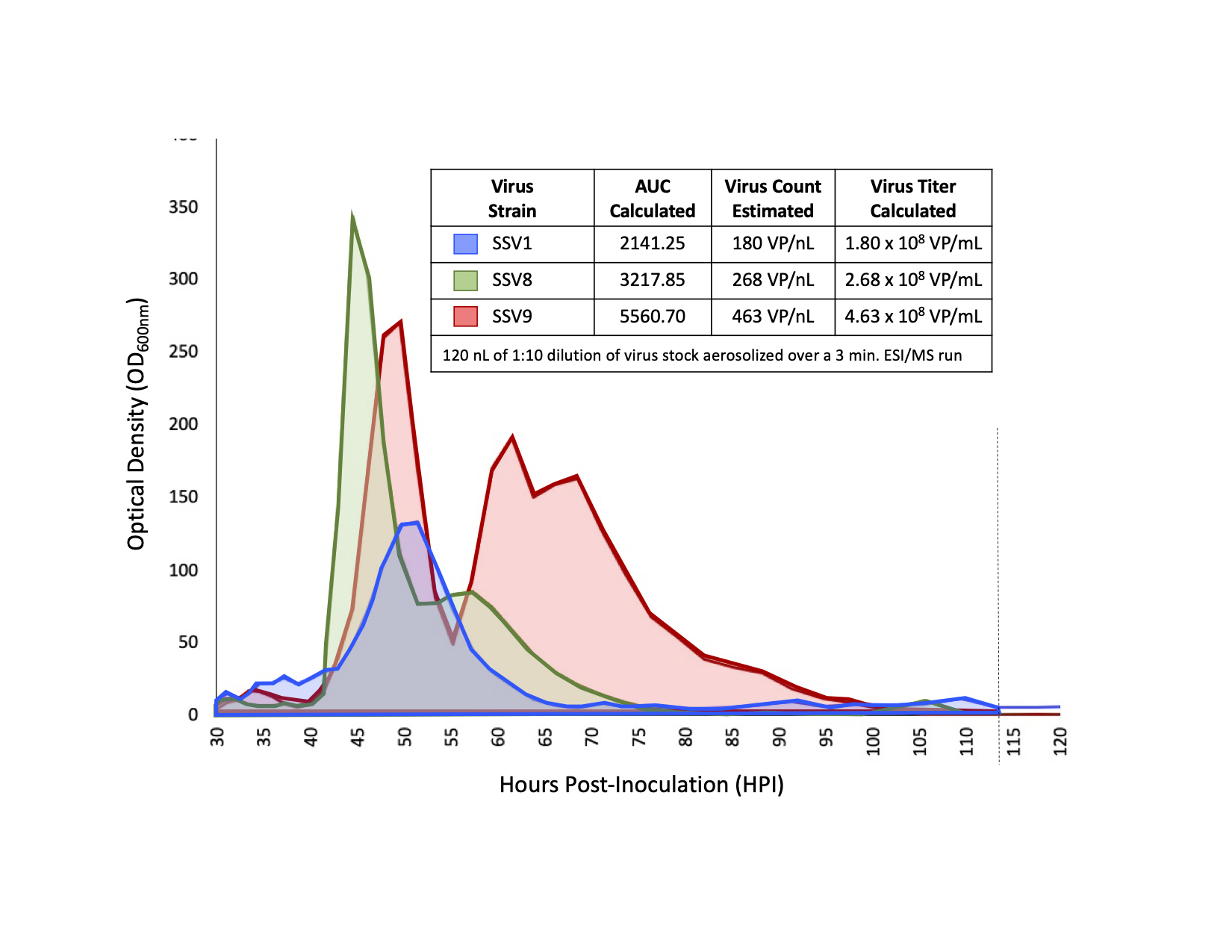

Supplement: Figure S1 — SSV titers via electrospray ionization mass spectrometry (ESI/MS). Concentrated viral suspension is diluted 1:10 and mixed with ionization solution (see section Methods). SSV particle count in 120 nL of suspension is monitored by a particle detector for 3 min. The area-under-the-curve (AUC) provides an estimate of titers. Each SSV has a characteristic spectrum. SSV1 (blue) is generally a single broad peak. SSV8 (green) exhibits a large single peak with a shoulder. SSV9 (red) typically presents as a double-peak. Since ESI/MS measures are based on mass:charge ratios of spherical particles and SSVs are fusiform (quasi-elliptical) shoulders and double peaks may be due to different orientations between aerosolization and triggering the detector. Alternatively, morphologically different populations may be present, including a sub-population of defective virus-like particles such as those that have lost their tails, which generate a shoulder or second peak. [file Image_1.jpeg]

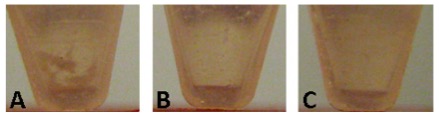

Supplement: Figure S2 — Cellular Debris in SSV9-infected but not in SSV1-infected Small-scaled Cultures. (A) SSV9-infected Sulfolobus strain Gθ has cell debris visible at 22 HPI; whereas, (B) SSV1-infected Gθ at 22 HPI, and (C) uninfected Gθ uninfected control do not. Cultures were allowed to settle overnight. Both infections were conducted at an MOI = 3. Cell debris is another line of evidence suggesting that SSV9 induces host cell lysis. [file Image_2.jpeg]

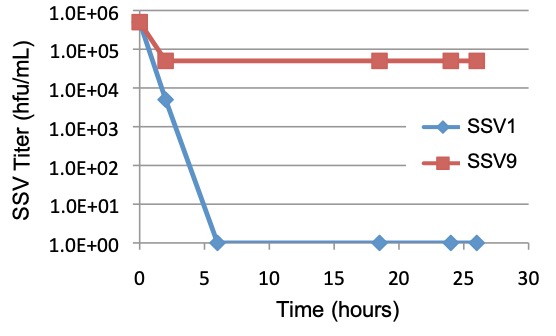

Supplement: Figure S3 — SSV viability after exposure to culture environment. At 75°C (pH 3.2) SSV9 (red) retains viability for up to 25 h; while titers of SSV1 (blue) decrease rapidly after 6 h in the same high temperature acid conditions. Plate-based serial dilution plaque-like “halo” assays (hfu/mL) were used to determine titers for each time point for SSV9 and SSV1. SSV9 virus particles (red) have greater stability in the high temperature and acid environment. [file Image_3.jpeg]

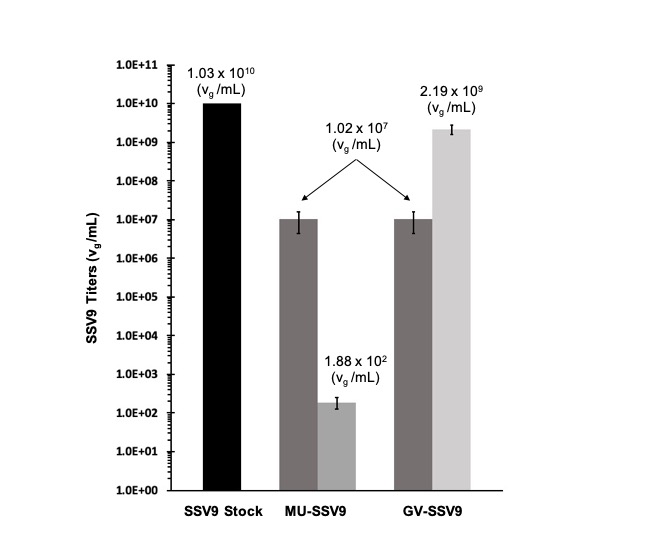

Supplement: Figure S4 — SSV9 genome abundance via quantitative polymerase chain reaction (qPCR). Viral genome counts were measured via qPCR after infections of Sulfolobus sp. strains MU and GV with SSV9. Liquid cultures of Sulfolobus sp. strains MU and GV were infected with SSV9 at an MOI = 0.1 (dark grey bars) from a SSV stock titered at 1.03 × 10^10^ vg/mL (black bar) followed by incubation for 72 h at 78°C (90 RPM shaking). At 72 HPI, titers were determined by qPCR from culture supernatant. SSV9 genomes per mL in MU culture dropped five orders of magnitude to within the lowest detection limit (medium grey bar), which indicates no productive infection. SSV9 genomes in GV culture increased by two orders of magnitude (light grey bar), indicating that Sulfolobus sp. strain GV is permissive to SSV9 replication. [file Image_4.jpeg]
