# Supplementary material for: Host-Dependent Differences in Replication Strategy of the Sulfolobus Spindle-Shaped Virus Strain SSV9 (a.k.a., SSVK1): Infection Profiles in Hosts of the Family Sulfolobaceae
Source: Front Microbiol. 2020 Jul 14;11:1218. doi: 10.3389/fmicb.2020.01218 (PMC7372142; doi:10.3389/fmicb.2020.01218)
Supplement: Table S1 — Percent inhibition calculations for SSV-G infection: Figure 4. [file Table_1.DOCX]

| **Table S1** – Percent Inhibition Calculations for SSV-Gθ Infection: Figure 4. | | | | |
| --- | --- | --- | --- | --- |
| **Trial** | **AUC_Riemann_** | **AUC_Gompertz_** | **PI (%)** | **R^2^_Gompertz_** |
| Gθ-CTL_UNINFECTED_ | 61.851 | 62.15 | 0 | 0.975 |
| Gθ-SSV1 | 56.454 | 56.83 | 8.6 | 0.988 |
| Gθ-SSV2* | 43.017 | 43.19 | 30.5 | 0.968 |
| Gθ-SSV3* | 42.972 | 43.42 | 30.1 | 0.951 |
| Gθ-SSV8 | 44.274 | 44.47 | 28.4 | 0.978 |
| Gθ-SSV10 | 51.57 | 51.97 | 16.4 | 0.9242 |
| * AUC values for SSV2 and SSV3 are underestimated since the curves had not reached stationary phase by the end of the trial time period; therefore, direct comparisons of PI are not appropriate. SSV2 and SSV3 show a delay in reaching stationary phase (i.e., *N_asymptote_*) when compared to SSV1 and SSV8. Extrapolated values predict that PI for SSV2 and SSV3 would be approximately equal to that shown for SSV1. | | | | |
