# Supplementary material for: Host-Dependent Differences in Replication Strategy of the Sulfolobus Spindle-Shaped Virus Strain SSV9 (a.k.a., SSVK1): Infection Profiles in Hosts of the Family Sulfolobaceae
Source: Front Microbiol. 2020 Jul 14;11:1218. doi: 10.3389/fmicb.2020.01218 (PMC7372142; doi:10.3389/fmicb.2020.01218)
Supplement: Table S2 — Percent inhibition for SSV infections on allopatric hosts: Figure 5. [file Table_2.DOCX]

| **Table S2** - Percent Inhibition for SSV Infections on Allopatric Hosts: Figure 5. | | | | |
| --- | --- | --- | --- | --- |
| **Trial** | **AUC** | **PI (%)** | **R^2^_Gompertz_** | **SE_AUC_ (±)** |
| S200-CTL_AVG_ | 113.4 | 0 | 0.978 | 0.792 |
| S200-SSV1_AVG_ | 107.5 | 5.3 | 0.986 | 4.104 |
| S200-SSV8_AVG_ | 75.0 | 33.9 | 0.965 | 0.691 |
| S200-SSV9_AVG_ | 31.4 | 72.4 | **0.301** | 1.332 |
|  |  |  |  |  |
| S444-CTL_AVG_ | 70.3 | 0 | 0.976 | 1.778 |
| S444-SSV1_AVG_ | 59.6 | 15.1 | 0.980 | 1.070 |
| S444-SSV8_AVG_ | 51.8 | 26.3 | 0.970 | 0.357 |
| S444-SSV9_AVG_ | 24.2 | 65.6 | **0.479** | 1.172 |
|  |  |  |  |  |
| S437-CTL_AVG_ | 102.6 | 0 | 0.962 | 0.221 |
| S437-SSV1_AVG_ | 99.0 | 3.5 | 0.981 | 1.603 |
| S437-SSV8_AVG_ | 69.9 | 31.8 | 0.969 | 6.272 |
| S437-SSV9_AVG_ | 31.6 | 69.2 | **0.531** | 4.728 |
| Coefficients of Determination (R^2^) less than 0.8 (in **bold**) are considered “failed” fits to the Gompertz model. For strain descriptions and references see main text. | | | | |
