# Supplementary material for: Host-Dependent Differences in Replication Strategy of the Sulfolobus Spindle-Shaped Virus Strain SSV9 (a.k.a., SSVK1): Infection Profiles in Hosts of the Family Sulfolobaceae
Source: Front Microbiol. 2020 Jul 14;11:1218. doi: 10.3389/fmicb.2020.01218 (PMC7372142; doi:10.3389/fmicb.2020.01218)
Supplement: Table S3 — Percent inhibition for SSV9 challenges on sympatric sulfolobales: Figure 8. [file Table_3.DOCX]

| **Table S3** - Percent Inhibition for SSV9 Challenges on Sympatric Sulfolobales: Figure 8. | | | | |
| --- | --- | --- | --- | --- |
| **Trial** | **AUC** | **PI (%)** | **R^2^_Gompertz_** | **SE_AUC_ (±)** |
| MU-CTL_1_ | 54.1 | -1.5 | 0.935 |  |
| MU-CTL_2_ | 51.5 | 3.5 | 0.857 |  |
| MU-CTL_3_ | 54.4 | -1.9 | 0.924 |  |
| MU-CTL_AVG_ | 53.3 | 0 | 0.920 | 0.92 |
|  |  |  |  |  |
| MU-SSV9_1_ | 53.3 | 0.0 | 0.931 |  |
| MU-SSV9_2_ | 54.5 | -2.2 | 0.913 |  |
| MU-SSV9_3_ | 55.8 | -4.7 | 0.941 |  |
| MU-SSV9_AVG_ | 54.5 | -2.3 | 0.942 | 0.72 |
|  |  |  |  |  |
| GV-CTL_1_ | 42.7 | 1.0 | 0.917 |  |
| GV-CTL_2_ | 39.9 | 7.3 | 0.946 |  |
| GV-CTL_3_ | 46.7 | -8.3 | 0.955 |  |
| GV-CTL_AVG_ | 43.1 | 0 | 0.967 | 1.97 |
|  |  |  |  |  |
| GV-SSV9_1_ | 31.8 | **26.3** | 0.912 |  |
| GV-SSV9_2_ | 31.4 | **27.2** | 0.960 |  |
| GV-SSV9_3_ | 30.4 | **29.6** | 0.843 |  |
| GV-SSV9_AVG_ | 31.2 | **27.7** | 0.934 | 0.41 |
| For strain descriptions and references see main text. | | | | |
